# Performance Evaluation of a Dengue IgG Rapid Diagnostic Test Designed to Determine Dengue Serostatus as Part of Prevaccination Screening

**DOI:** 10.1128/spectrum.00711-21

**Published:** 2022-05-23

**Authors:** Vasco Liberal, Remi Forrat, Cong Zhang, Charles Pan, Matthew Bonaparte, Wushan Yin, Lingyi Zheng, Valeria Viscardi, Yukun Wu, Yasemin Ataman-Önal, Stephen Savarino, Catherine Chen

**Affiliations:** a CTK Biotech, Poway, California, USA; b Sanofi, Marcy l’Etoile, France; c Beijing Genesee Biotech, Beijing, China; d Sanofi, Swiftwater, Pennsylvania, USA; Center for Research and Advanced Studies (CINVESTAV-IPN)

**Keywords:** dengue, diagnostic test, immunization

## Abstract

The World Health Organization has recommended prevaccination screening for prior dengue infection as the preferred approach prior to vaccination with the dengue vaccine CYD-TDV. These screening tests need to be highly specific and sensitive, and deliverable at the point-of-care. We evaluate here the sensitivity and specificity of the newly developed *OnSite* Dengue IgG rapid diagnostic test (RDT). A retrospective double-blind study of the sensitivity and specificity of the *OnSite* Dengue IgG RDT was performed using a sample panel consisting of archived serum specimens collected during CYD-TDV clinical trials in Latin American and Asia, with the reference serostatus for each sample determined by an algorithm using measured dengue PRNT_90_, PRNT_50_, and NS1 IgG ELISA. An additional panel of dengue seronegative samples positive for other flaviviruses and infections was used to assess cross-reactivity. Samples were included from 579 participants; 346 in the specificity panel and 233 in the sensitivity panel. The *OnSite* dengue IgG RDT exhibited a specificity of 98.0% (95% CI = 95.9 to 99.2) and sensitivity of 95.3% (95% CI = 91.7 to 97.6). The sensitivity for samples exhibiting a multitypic immune profile (PRNT_90_-positive to >1 dengue serotype) was 98.8% while for monotypic immune samples (PRNT_90_-positive to a single dengue serotype) it was 88.1%. The *OnSite* dengue IgG RDT showed minimal to no cross-reactivity to related flaviviruses. These findings support the use of the *OnSite* dengue IgG RDT to determine dengue serostatus in CYD-TDV prevaccination screening.

**IMPORTANCE** Dengue remains a significant public health issue, with over 5.2 million cases reported to the World Health Organization (WHO) in 2019. The tetravalent dengue vaccine (CYD-TDV) is currently licensed for use in those aged ≥9 years; however, vaccinees with no previous exposure to dengue experience an increased risk of hospitalized and severe dengue upon subsequent heterotypic infection. Consequently, WHO recommends screening for prior dengue infection before vaccination. Screening tests for previous infection need to be highly specific and sensitive, and deliverable at the point-of-care. High sensitivity ensures that the largest number of individuals with previous infection can be identified and vaccinated, while high specificity prevents the inadvertent vaccination of those without previous infection. This study of the OnSite Dengue IgG Rapid Test, which was explicitly developed to meet this need, found that it had both high specificity (98.0% [95% CI = 95.9 to 99.2]) and sensitivity (95.3% [95% CI = 91.7 to 97.6]).

## INTRODUCTION

Dengue is a mosquito-borne virus transmitted by the female Aedes aegypti and Aedes albopictus species; there are four distinct, but closely related, serotypes that cause dengue disease (serotypes 1–4). Dengue remains a significant public health issue, with over 5.2 million cases reported to the World Health Organization (WHO) in 2019 (1), with over 3 million cases in the Americas alone (2). The tetravalent dengue vaccine (CYD-TDV; Dengvaxia, Sanofi) was first approved for dengue prevention in 2015. As of October 2021, the vaccine is licensed for use in individuals aged ≥9 years in 22 countries as well as the European Economic Area, and in one country for those ≥12 years of age.

A recent case-cohort study re-examined samples and data obtained from three CYD-TDV efficacy studies, and demonstrated that CYD-TDV vaccination conferred robust protection against hospitalized and severe dengue illness over 5 years in baseline dengue-seropositive participants, while an increased risk of these outcomes was observed in baseline dengue seronegative participants during the 5-year follow-up period (3). As a result of these findings, WHO recommends that only individuals with evidence of prior dengue infection are vaccinated (4). Individuals with no documented laboratory-confirmed dengue infection will need to have their dengue serostatus determined through dengue serological assays to identify individuals eligible for vaccination (4). Suitable tests should exhibit very high specificity, with minimal cross-reactivity to other flaviviruses, and high sensitivity in determining prior infections with each of the four dengue serotypes. High sensitivity is required to ensure that the largest number of seropositive individuals can be identified and vaccinated, while high specificity is needed to prevent the inadvertent vaccination of seronegative individuals (5). Furthermore, tests which can be administered at point-of-care would be preferred to streamline testing and the subsequent vaccination of eligible individuals.

Anti-dengue IgG enzyme-linked immunosorbent assay (ELISA) tests are indicated for determining prior dengue infection status and are routinely used in serosurveys to characterize dengue incidence. Recent studies have found that commercially available IgG ELISAs exhibit high sensitivity and specificity, but typically display meaningful levels of cross-reactivity to other flaviviruses (6–8). In addition, ELISAs are time-consuming and require significant laboratory infrastructure. In contrast, rapid diagnostic tests (RDTs), which can be performed at the point-of-care, require minimal training and provide results more rapidly than traditional ELISA or neutralization tests, could enable rapid, simple screening in regions where dengue is endemic, which are often resource-limited and lack the laboratory capacity to perform ELISAs (9). Commercially available RDTs detecting anti-dengue IgG display high specificity and low flavivirus cross-reactivity in identifying prior dengue infection, but are suboptimal in sensitivity (6–8). Recent findings for new point-of-care dengue immunoassays developed for prevaccination screening indicated higher sensitivity than existing commercial dengue IgG-containing RDTs, but suboptimal specificity (10–12).

The body of available data underscores the need for new point-of-care tests that determine prior dengue infection status with both high specificity and sensitivity. The *OnSite* Dengue IgG Rapid Test (CTK Biotech, Poway, CA, USA) is a lateral flow chromatographic immunoassay explicitly developed to meet this need and differs from the *OnSite* Dengue IgG/IgM Combo Rapid Test evaluated in previous studies (7, 8), which is intended to detect active or recent dengue infection. The *OnSite* Dengue IgG Rapid Test exclusively detects IgG and is intended to detect past exposure to the dengue virus. Here, we report its performance in determining prior dengue infection status.

## RESULTS

A total of 581 samples were tested from the immunogenicity subsets of CYD14 and CYD15. The specificity panel consisted of 346 samples, the majority of which (84%) were from the CYD15 study, and almost half (46%) came from participants from Mexico (Table 1); two samples were removed from the specificity panel as one was later confirmed as seropositive and one had a missing PRNT result. The sensitivity panel consisted of 233 samples, the majority of which (73%) were from CYD15, and almost half (47%) came from participants from Colombia (Table 1).

**TABLE 1:** Baseline characteristics of participants contributing to the specificity and sensitivity panels

Characteristic, *n* (%)	Specificity panel (*n* = 346)	Sensitivity panel (*n* = 233)
Study		
CYD14	57 (16)	64 (27)
CYD15	289 (84)	169 (73)
Country		
Philippines	57 (16)	64 (27)
Colombia	57 (16)	110 (47)
Honduras	29 (8)	24 (10)
Mexico	160 (46)	23 (10)
Puerto Rico	43 (12)	12 (5)
Age		
6 to 8 yrs	27 (8)	17 (7)
9 to 16 yrs	319 (92)	216 (93)

The reference algorithm classified participants contributing samples to the performance study panel into six groups, according to the reference dengue PRNT_90_, PRNT_50_, and NS1 IgG ELISA results (Table S1), and were further subdivided into those who were reference seropositive and reference seronegative for the sensitivity and specificity assessments. A large majority of participants were classified into two of the groups: reference group 1, participants with negative PRNT_90_, PRNT_50_, and NS1 IgG ELISA results (254/579; 43.9%) and reference group 6 those with positive PRNT_90_ test results, regardless of the NS1 IgG ELISA results (231/579; 39.9%).

The *OnSite* dengue IgG RDT exhibited a specificity of 98.0% (95% CI = 95.9 to 99.2) and sensitivity of 95.3% (95% CI = 91.7 to 97.6). The sensitivity for samples exhibiting a multitypic immune profile was 98.8%, while for monotypic immune samples it was 88.1%, with detection by individual serotype ranging between 82% and 94% (Table 2). Similarly, the *OnSite* dengue IgG RDT exhibited high sensitivity in samples from individuals with both recent (96.6%; 95% CI = 90.4 to 99.3) and remote virologically confirmed dengue (VCD) (94.4%; 95% CI = 87.5 to 98.2).

**TABLE 2:** Sensitivity of the *OnSite* dengue IgG RDT by dengue immune profile^*a*^

Profile^*b*^	*N*	*n*	Sensitivity, %
Multitypic	171	169	98.8
Monotypic (all)	59	52	88.1
DENV-1	11	9	81.8
DENV-2	24	21	87.5
DENV-3	17	16	94.1
DENV-4	7	6	85.7

a N, number of dengue seropositive samples by PRNT_90_ (reference seropositive group 6); n, number of dengue seropositive samples by *OnSite* dengue IgG RDT.

b Multitypic immune, neutralizing antibodies ≥10 (1/dil) against ≥2 dengue serotypes by PRNT_90_; monotypic immune, neutralizing antibodies ≥10 (1/dil) against only 1 dengue serotype by PRNT_90_.

Based on the sensitivity and specificity estimates for the RDT, the corresponding positive and negative predictive values (PPV, NPV) and false discovery and omission rates (FDR, FOR) as a function of dengue seroprevalence are shown in Table S2. In settings where the seroprevalence is ≥20%, the positive predictive value exceeds 90%, corresponding to a FDR <10%, the proportion of test-positive individuals that would be false positives. The negative predictive value is at or above 90% where dengue seroprevalence is ≤70%, corresponding to a FOR of ≤10%.

The *OnSite* dengue IgG RDT showed minimal to no cross-reactivity to samples obtained from individuals with prior exposure to non-dengue flaviviruses (Table 3). In the reference seronegative samples from CYD14 and CYD15 that were positive for Yellow fever virus (YFV) and Japanese encephalitis virus (JEV), one YFV-positive sample (1.4%) was a false dengue-positive, with no cross-reactivity in JEV-positive samples. In the additional cross-reactivity testing, there was minimal cross-reactivity with YFV-positive and JEV-positive samples, 1/42 (2.4%) and 1/36 (2.8%), respectively, and no cross-reactivity to Zika. Similarly, there was minimal to no cross-reactivity to samples with other potential sources of cross-reactivity (Table 4).

**TABLE 3:** Cross-reactivity of the *OnSite* dengue IgG RDT with samples positive for related flaviviruses and dengue seronegative by the reference algorithm^*a*^

Samples	Flavivirus	*N*	Cross-reactivity, *n* (%)
CYD14 CYD15	YFV	72	1 (1.4)
	JEV	5	0 (0)
Additional cross-reactivity panel	Zika	35	0 (0)
	YFV	42	1 (2.4)
	JEV	36	1 (2.8)
	WNV	32	0 (0)

a N, number of samples flavivirus positive and dengue reference seronegative; n, number of samples dengue seropositive by *OnSite* dengue IgG RDT; JEV, Japanese encephalitis virus; WNV, West Nile virus; YFV, yellow fever virus.

**TABLE 4:** Cross-reactivity of the *Onsite* dengue IgG RDT for other diseases or special conditions in the additional cross-reactivity panel

Disease/condition	*N*	Cross-reactivity, *n* (%)
Antinuclear antibodies	13	0 (0)
Borrelia (Lyme)	12	0 (0)
Chikungunya	19	0 (0)
Cytomegalovirus	12	0 (0)
SARS-CoV-2	36	1 (2.8)
Epstein-Barr virus	13	0 (0)
Enterovirus	15	0 (0)
Human anti-murine antibody	15	0 (0)
Hepatitis A	11	0 (0)
Hepatitis B	16	0 (0)
Hepatitis C	18	0 (0)
Human immunodeficiency virus 1/2	36	1 (2.8)
Herpes simplex virus type 1	10	0 (0)
Herpes simplex virus type 2	11	0 (0)
Influenza A	17	0 (0)
Influenza B	15	0 (0)
Leptospirosis	12	1 (8.3)
Malaria	20	0 (0)
Measles	23	0 (0)
Parvovirus B19	12	0 (0)
Rheumatoid Factor	14	0 (0)
Rubella	23	1 (4.3)
Syphilis	12	0 (0)
Varicella-zoster virus	12	1 (8.3)

## DISCUSSION

The *OnSite* dengue IgG RDT fills a diagnostic gap for a simple, highly accurate dengue immunoassay that can be used at point-of-care as part of prevaccination screening for dengue serostatus before administering CYD-TDV, as recommended by WHO (4) and in conformance with the optimal test performance characteristics recently proposed for this diagnostic indication (13). The test displayed very high specificity and minimal-to-no cross-reactivity to any of the diseases or conditions tested. Compared with currently marketed dengue IgG/IgM combination RDTs, the *OnSite* dengue IgG RDT has a higher sensitivity for identifying prior dengue infection, which is comparable to that of marketed IgG ELISAs, whose performance in identifying prior dengue infection has been carefully assessed (6–9). Moreover, the *OnSite* dengue IgG RDT shows considerably higher specificity and sensitivity than other point-of-care tests explicitly developed for identifying prior dengue infection, especially for dengue monotypic immune individuals where the other newer tests exhibit sensitivity of 51% to 67% (11, 14).

The benefit/risk of a prevaccination screening test is dependent on performance characteristics as well as positive and negative predictive values, which vary with the prevalence of prior dengue infection in a given population. Flasche et al. (15) and Rodriguez-Barraquer et al. (5) have proposed that the range over which a dengue screening test is safely “fit for purpose” is the span over which PPV exceeds 90% and NPV exceeds 75%, in the case of this new RDT from 16% to 87% dengue seroprevalence (see Table S2). Fongwen et al. have proposed that under optimal circumstances, both PPV and NPV would be ≥90%, where the false discovery rate and false omission rate would both be <10% (13). For the *OnSite* Dengue IgG Rapid Test, this would correspond to dengue seroprevalence spanning 16% to 69%. While the benefit-risk calculus may vary for different settings and policy priorities, this new RDT offers favorable performance in settings where a range from low-moderate to high dengue seroprevalence prevails.

In addition to the very high specificity and sensitivity, this novel test exhibited minimal cross-reactivity in sera obtained from people previously exposed to non-dengue flaviviruses and other relevant diseases and potential sources of cross-reactivity. Cross-reactivity can be triggered in patients with previous exposure to non-dengue flaviviruses and development of antibodies which are cross-reactive to the related dengue antigens, as well as by structural similarity between dengue antigens used by dengue diagnostic tests and other viral antigens (16, 17). There was no cross-reactivity observed in prior Zika-exposed people, and only nominal cross-reactivity to other disease states, suggesting application of this test will be useful in many dengue-endemic regions where Zika, YFV, and JEV are prevalent. In a recent report by Lustig et al., samples taken during the acute phase of a documented, symptomatic, SARS-CoV-2 infection displayed notable cross-reactivity (22%) using a dengue IgG/IgM lateral-flow RDT, but not with dengue IgG and IgM ELISAs (17). However, in contrast to the Lustig et al. study that used acute samples from symptomatic individuals, in the present study convalescent SARS-CoV-2 samples were used to evaluate cross-reactivity (3 weeks to 3 months after lab-confirmed SARS-CoV-2 infection). Minimal cross-reactivity to SARS-CoV-2 was observed (1/36; 2.8%). As was discussed in the paper by Lustig et al. (17), consideration should be given during the ongoing SARS-CoV-2 pandemic to avoid potential cross-reactivity as a risk mitigation step if assessing individuals for prior dengue exposure before CYD-TDV vaccination. As the pandemic evolves, continued monitoring of dengue IgG RDT cross-reactivity with SARS-CoV-2 pre-immune sera will be important.

The *OnSite* dengue IgG Rapid Test displays just over 88% sensitivity in identifying dengue monotypic immune individuals, and in a head-to-head comparison was comparable to two of the more sensitive, commercially available dengue IgG ELISAs (18). Achieving high sensitivity in detecting true dengue-seropositive individuals is key to reducing the population risk of severe dengue with CYD-TDV vaccination in low-moderate to high dengue endemic settings. As individuals who have experienced a single prior dengue infection are at greatest risk of more severe disease upon subsequent natural exposure to heterotypic dengue (19), the high sensitivity of this RDT in detecting those with a monotypic immune profile, a proxy for this risk group, is likely to enhance benefit both at the individual and population level by accurately and efficiently identifying this subgroup as candidates for vaccination.

All currently available dengue RDTs were designed to detect active dengue infection and are, therefore, not optimized for detecting prior infection (9). While there is evidence indicating that anti-dengue IgG antibodies persist for decades after infection (20), it is reasonable to question how long after exposure the IgG RDT is able to detect a prior dengue infection. We have shown that in dengue-endemic settings, the *OnSite* dengue IgG RDT remains highly sensitive in identifying children who experienced documented symptomatic dengue infection up to 5 years prior to sample collection. This may inform the planning of dengue vaccine implementation programs using a screen-and-vaccinate strategy, where periodic screening at regular intervals has been considered (21).

This study has limitations. The study population may not be fully representative of the underlying population from which the samples were selected, where true randomization was affected by the requirement for additional informed consent and a lack of sufficient volume of samples. Furthermore, samples were restricted to a subset of five out of the 10 countries participating in the phase III CYD-TDV vaccine trials due to country-level restrictions on future research use of serum samples. However, while this study did not use a truly random selection of samples, these results are comparable to a study of the sensitivity and specificity of the *OnSite* dengue IgG RDT when samples from subjects ≥6 years of age of the entire immunogenicity subset of CYD14 and CYD15 are used; sensitivity (91.8%; 95% CI = 90.7 to 92.9) and specificity (96.1%; 95% CI = 94.5 to 97.3) (18). Additionally, the study population does not fully reflect the exposure history of individuals currently living in dengue endemic areas, as the samples used in this study were drawn in 2011 to 2012, prior to the large Zika epidemic in the Americas (2015 to 2017) and before the ongoing SARS-CoV-2 pandemic (starting end 2019). This may impact test performance, although cross-reactivity results suggest that screening of individuals in settings with appreciable Zika seroprevalence is unlikely to increase the false-positive rate. Corroborative studies to evaluate performance of the *OnSite* dengue IgG RDT in populations previously exposed to Zika are under way to expand on the findings presented here. The prudence of excluding Zika false positivity is underscored by evidence that a prior Zika infection is associated with an increased risk of symptomatic and severe dengue (22), although this does not necessarily predict such an increase in risk for dengue naive, Zika-exposed individuals following CYD-TDV vaccination.

The *OnSite* dengue IgG rapid test detects anti-dengue virus IgG in human serum, with high sensitivity, very high specificity and minimal-to-no cross-reactivity with related flaviviruses. It can be performed rapidly by minimally skilled personnel and without the use of laboratory equipment. These findings offer a favorable benefit-risk ratio in using the *OnSite* dengue IgG rapid test for screening for true dengue seropositive participants for vaccination with CYD-TDV.

## MATERIALS AND METHODS

### Study design and samples.

This was a retrospective observer-blind study conducted to determine the sensitivity and specificity of the *OnSite* Dengue IgG RDT using archived serum samples collected at baseline from 6- to 16-year-old participants from the two pivotal CYD-TDV vaccine Phase III clinical trials, CYD14 (NCT01373281) and CYD15 (NCT01374516), conducted in Asia and Latin America, respectively (23, 24). Participants in these studies were asked for consent for future use of their samples as part of the original enrollment in the trials (NCT01134263 and NCT01187433), and samples from those who did not provide this consent were not included in this analysis, which did not require additional IRB approval. This study observed the principles of Good Clinical Laboratory Practice (GCLP).

The reference dengue serostatus of CYD14 and CYD15 participants prior to vaccination at baseline was determined using a previously published comparator algorithm that is based on the composite results of the dengue plaque reduction neutralization test at 50% (PRNT_50_), and 90% (PRNT_90_), and the dengue NS1 IgG ELISA (Table S1) (6–8). All baseline samples from participants classified as reference dengue seronegative from the immunogenicity subsets of CYD14 and CYD15, who had provided informed consent, and for which there was sufficient volume for testing, were included for assessment of RDT specificity. A random subset of samples was selected from all reference dengue seropositive participants to assess the sensitivity of the RDT; participants who did not provide additional informed consent for future research use of their samples, and those with insufficient volume for testing, were then removed from the selected sensitivity subset.

Samples forming an additional cross-reactivity panel were also tested by the *OnSite* Dengue IgG RDT. Cross-reactivity was assessed in samples that were dengue seronegative from individuals with prior Zika, JEV, YFV, and West Nile virus (WNV) exposure (details in Supplementary Methods). Further samples from patients exposed to additional pathogens and with special conditions were obtained from commercial sources (ABO Pharmaceuticals, San Diego, CA; Discovery Life Sciences, San Luis Obispo, CA; Access Biologicals, Vista, CA; Boca Biolistics, Pompano Beach, FL; NY Biologics, New York, NY; Eurofins Biomnis, Lyon, France). This panel includes common infectious diseases, pathogens that co-circulate in the same geographic regions as dengue, and special conditions resulting from immune system dysregulation.

### Test procedures.

The *OnSite* Dengue IgG Rapid Test (CTK Biotech, San Diego, USA) is a new CE-marked lateral flow chromatographic immunoassay which qualitatively detects dengue IgG with a test strip consisting of a burgundy colored conjugate pad containing four recombinant dengue envelope antigens, representing each dengue serotype, conjugated with colloidal gold. Equilibration of serum specimens and assay components was performed at room temperature (15 to 30°C). Approximately 5 μL of specimen was transferred to the sample well on the cassette using capillary tubes in the R0065C test kit which was immediately followed by addition of two drops (90 to 120 μL) of sample diluent to the sample diluent well. Results were recorded at 20 to 25 min following this and a photographic record of test devices taken. Any tests which did not develop an internal control line were classified as invalid and were repeated with an additional test device, with invalid results recorded but not used for the determination of test performance characteristics.

The clinical performance evaluation study was initiated and completed between June 2020 to October 2020, including specimen and materials shipment, testing, data analysis, and report generation. The study was conducted internally at the CTK Biotech facilities in Poway, California and was performed over multiple days by four operators with blinded samples. Cross-reactivity studies were conducted contemporaneous to the clinical performance studies at the CTK Biotech facilities in Poway, CA. Assessment of sensitivity by time since infection was conducted at the Global Clinical Immunology Laboratory, Sanofi, Swiftwater, PA.

### Outcomes.

The primary objective of the clinical performance evaluation study was to determine the clinical sensitivity and specificity of the *OnSite* dengue IgG RDT in identifying prior dengue infection. Exploratory objectives were an estimation of test sensitivity by dengue immune profile (where multitypic immune is defined by neutralizing antibodies ≥10 [1/dil] against ≥2 dengue serotypes by PRNT_90_ and monotypic immune by neutralizing antibodies ≥10 [1/dil] against only 1 dengue serotype by PRNT_90_), and by serotype for samples classified as monotypic immune.

Additional objectives of the study were to evaluate cross-reactivity to the related flaviviruses (Zika, WNV, YFV, and JEV), as well as other related, common, or co-circulating pathogens and conditions; and to estimate the test sensitivity in those with a symptomatic VCD infection 1 to 13 months (“recent”) and 2.5–≤5 years (“remote”) prior to sample collection.

### Sensitivity assessment by time since symptomatic dengue infection.

To assess sensitivity of the assay by time since a documented, symptomatic dengue infection, placebo recipients in the CYD14 and CYD15 Phase III trials who had a virologically confirmed, symptomatic dengue infection during the first 12 months of the studies (months 0 to 12) were identified. Archived serum samples obtained at month 13 and at approximately month 48 from these participants were used to assess the sensitivity of the RDT after a recent and a remote infection, respectively (7) (Fig. S1).

### Statistical analysis.

Sample size for the clinical performance study was calculated to achieve an overall power >90% to demonstrate both specificity of 99% that excludes a lower limit (LL) of the 95% confidence interval (CI) of 95%, and sensitivity of 85% that excludes a LL95% CI >75% in the 6- to 16-years-old age group. Following these calculations, the total number of samples determined was of 362 dengue IgG seronegatives (power of 99.6%) and 211 dengue IgG seropositives (power of 95.2%), for an overall study power of 94.8%, and a total of 573 samples tested.

The sensitivity and specificity of the *OnSite* Dengue IgG RDT was derived using the reference algorithm as a comparator (8). Immunoassay “true” seropositive samples were those determined as seropositive by both immunoassay and reference algorithm, and immunoassay “true” seronegative samples were those which were determined as seronegative by both the immunoassay and reference algorithm. The respective 95% CI were calculated using the Clopper-Pearson method. Cross-reactivity was calculated as the percentage of samples from dengue-naive individuals with a positive RDT result over the total number of samples for each non-dengue infectious agent or special condition.

### Data availability.

Full diagnostic data from this study can be found in the supplementary appendix.
