# Supplementary material for: Performance Evaluation of a Dengue IgG Rapid Diagnostic Test Designed to Determine Dengue Serostatus as Part of Prevaccination Screening
Source: Microbiol Spectr. 2022 May 23;10(3):e00711-21. doi: 10.1128/spectrum.00711-21 (PMC9241662; doi:10.1128/spectrum.00711-21)

Supplemental Materials

Supplemental Methods

Additional flavivirus cross-reactivity panel

Cross-reactivity to Zika was determined in samples from individuals with prior Zika exposure obtained from several commercial and academic sources; including travelers from Israel, Spain and the US to Zika epidemic countries, and individuals participating in CYD15 as well as a cross-sectional surveillance study in Guatemala. Prior Zika exposure was confirmed in all individuals by Zika microneutralization titer ≥100 (1/dil) and by Zika RT-PCR in an acute serum sample in the majority of participants, and lack of prior dengue exposure was determined by dengue seronegative classification by the reference algorithm.

Japanese encephalitis virus (JEV) cross-reactivity was determined in samples obtained from two sources: US volunteer donors after receipt of an inactivated JE vaccine (JE-VAX®) who were negative by dengue $PRNT_{50}$; and pre-vaccination samples from participants in CYD14 who were JEV $PRNT_{50}$ positive (titer ≥10), and dengue seronegative by the dengue reference algorithm.

Yellow fever virus (YFV)-exposure positive samples were from two sources: US adult volunteers in a phase II study of CYD-TDV (CYD51; NCT01488890) who were vaccinated with an attenuated YFV vaccine (YF-VAX®) and who were classified as dengue seronegative by the dengue reference algorithm; and pre-vaccination samples from participants in CYD15 who were YFV $PRNT_{50}$ positive (titer ≥10), and dengue seronegative by the dengue reference algorithm.

Samples from individuals previously exposed to West Nile Virus (WNV) were obtained from US and Israeli adults who were WNV IgG positive (West Nile Virus DxSelect™ ELISA, Focus Diagnostics) and negative in the dengue NS1 IgG ELISA (<9 EU/ml).

Table S1. Baseline dengue serostatus of samples by the reference algorithm

Group	Reference test results			Interpretation	RDT01 panel, n (%)	
	$PRNT_{90}$[1]	$PRNT_{50}$[1]	Dengue NS1 IgG ELISA[2]		By group	By classification
**1**	Negative	Negative	Negative	Reference seronegative	254 (43.9%)	346
**2**	Negative	Positive	Negative		44 (7.6%)	
**3**	Negative	Negative	Low positive		48 (8.3%)	
**4**	Negative	Positive	Low positive	Reference seropositive	0 (0%)	233
**5**	Negative	Any[3]	High positive		2 (0.3%)	
**6**	Positive	Positive	Any[3]		231 (39.9%)	

[1] $PRNT_{50}$ and $PRNT_{90}$ positive is defined by titer ≥10 (1/dil) against ≥1 dengue serotype

[2] Dengue virus NS1 IgG ELISA results classified as negative (titer <9 EU/ml), low positive (≥9 to <50 EU/ml) and high positive (≥50 EU/ml)

[3]Any; positive or negative

ELISA, enzyme-linked immunosorbent assay; n, number of samples fulfilling criteria; PRNT, plaque reduction neutralization test.

Description of the NS1 IgG ELISA: Nascimento EJM, et al. J Virol Methods 2018;257:48-57.

Description of the PRNT assay: Timiryasova TM, et al. Am J Trop Med Hyg 2013;88:962-70.

Table S2. Predictive values and false detection and omission rates for the *OnSite* Dengue IgG RDT over a range of dengue seroprevalence rates.

**Dengue Prevalence, %**	**PPV, %**	**NPV, %**	**FDR[1], %**	**FOR[2], %**
15	89.4	99.2	10.6	0.8
20	92.3	98.8	7.7	1.2
25	94.1	98.4	5.9	1.6
30	95.3	98.0	4.7	2.0
35	96.2	97.5	3.8	2.5
40	96.9	96.9	3.1	3.1
45	97.5	96.2	2.5	3.8
50	97.9	95.4	2.1	4.6
55	98.3	94.5	1.7	5.5
60	98.6	93.3	1.4	6.7
65	98.9	91.8	1.1	8.2
70	99.1	89.9	0.9	10.1

[1]FDR, false detection rate (1-PPV).

[2]FOR, false omission rate (1-NPV).

Figure S1. Timeline for sample collection in placebo recipients from CYD14 and CYD15 who had a virologically confirmed, symptomatic dengue infection during Months 0–12, Month 13 samples for sensitivity for recent dengue infection detection and Month 48 samples for remote infection detection.

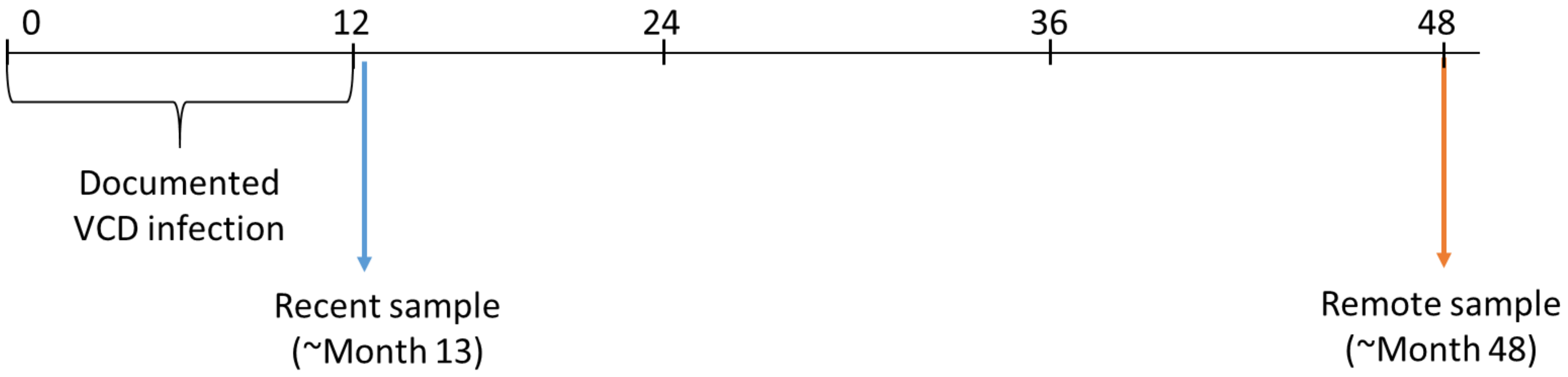

Supplement: SUPPLEMENTAL FILE 1 — Supplemental material. Download spectrum.00711-21-s001.pdf, PDF file, 0.2 MB [file spectrum.00711-21-s001.pdf]
